# Effects of consecutive days of matchplay on maximal hip abductor and adductor strength in female field hockey players

**DOI:** 10.1186/s13102-021-00394-x

**Published:** 2022-01-03

**Authors:** Violeta Sánchez-Migallón, Álvaro López-Samanes, Juan Del Coso, Archit Navandar, Per Aagaard, Víctor Moreno-Pérez

**Affiliations:** 1 Exercise Physiology Group, School of Physiotherapy, School of Health Sciences, Faculty of Health Sciences, Universidad Francisco de Vitoria, Pozuelo de Alarcón, 28223 Madrid, Spain; 2 Centre for Sport Studies, Rey Juan Carlos University, Fuenlabrada, Spain; 3 Faculty of Sport Sciences, Universidad Europea de Madrid, Villaviciosa de Odón, Spain; 4 Research Unit for Muscle Physiology and Biomechanics, Department of Sports Science and Clinical Biomechanics, University of Southern Denmark, Odense, Denmark; 5 Center for Translational Research in Physiotherapy, Department of Pathology and Surgery, Universidad Miguel Hernández, Elche, San Juan Spain

**Keywords:** Team sports, Risk factors, Consecutive matches, External load

## Abstract

**Background:**

The purpose of this study was to examine the effects of two competitive field hockey matches, played on consecutive days, on maximal isometric hip adductor and abductor strength, wellness and fatigue.

**Methods:**

Fourteen professional female field hockey players (age: 20.4 ± 5.4 years; body mass: 60.7 ± 7.2 kg; height: 167.0 ± 1.0 cm) volunteered to participate in this investigation. Maximal isometric hip adductor and abductor strength were obtained before (pre-match 1) and after the first match (post-match 1), after the second match (post-match 2), and 48 h after the second match. Locomotion patterns during the matches were obtained with portable Global Positioning System (GPS) and perceived exertion (RPE) was assessed after each match. In addition, Wellness Questionnaire (5-WQ) and the Total Quality Recovery Scale (TQR) were employed before the matches and 48 h after the second match.

**Results:**

For the non-dominant limb, the maximal isometric hip adductor and abductor strength were lower after post-match 2 when compared to pre-match 1 (*p* = 0.011). Hip abductor strength in the non-dominant limb remained reduced 48 h after post-match 2 (*p* < 0.001). There were no differences in the total distance covered when comparing match 1 and match 2. Players reported more acute fatigue (5-WQ, *p* = 0.009) and increased muscle soreness on pre-match 2 compared to pre-match 1 (*p* = 0.015), while fatigue returned to pre-competition levels 48 h after post-match 2 (*p* = 0.027). No changes were observed in the TQR.

**Conclusion:**

The assessment of maximal adductor and abductor strength before and after competitive matches, in addition to evaluating self-perceived fatigue by a wellness questionnaire can help to identify field hockey players with excessive fatigue responses during tournaments with a congested match program.

## Background

Groin injuries are one of the most frequent overuse injuries in field hockey, accounting for up to 10% of all injuries reported in competitive field hockey teams [1]. Preventing groin injury in field hockey is essential to reduce the burden on players and teams [2]. Thus, the implementation of preventive measures, the identification of the risk factors associated with the development of the injury, and the understanding of potential mechanisms exacerbating the severity of groin injury are paramount for this aim [3]. The complexity involved to ascertain the causes of groin injuries is well recognized, as in most cases the cause of injury is multifactorial [4]. However, previous studies conducted in other sports have identified several modifiable factors that affect the risk of groin injury, such as reduced flexibility [5, 6] and muscle weakness [7–11]. Amongst the modifiable risk factors, low levels of isometric hip adductor strength and/or imbalance in the hip adductor/abductor strength ratio have received much attention in the literature across a range of team sports [8]. However, the evidence remains somewhat conflicting and there is still no consensus regarding the role of low hip adductor/abductor muscle strength in the etiology of a groin injury.

Irrespective of the complexities of risk factors, field hockey match-play has been consistently associated with a several-fold greater incidence of muscle injuries when compared to training [1]. The combination of high physical demands during match play together with insufficient recovery time in between games may in part explain the higher injury rates reported during field hockey match play compared to training [12]. Field hockey match-play involves frequent explosive actions that require a high rate of force development (RFD) and significant eccentric muscle loadings, such as change of directions, accelerations, and decelerations [13].

Previous studies have shown that demands of match play in intermittent explosive sports such as football can induce muscle damage and post-match fatigue lasting up to 72 h, the latter due to a marked depletion in muscle glycogen concentrations [14]. Field hockey players are habitually exposed to densely packed match programs with a high number of matches performed in daily conjunction, particularly during international tournaments (e.g. Olympic Games) [15]. Previous reports have suggested that the accumulation of neuromuscular fatigue during successive matches may lead to an altered physical performance of the players [16]. In addition, the effects of a congested calendar in intermittent sports may also include reductions in maximal hip adductor strength [17].

Further, Crow et al. [6] reported that hip adductor muscle strength was reduced (− 6%) during the week that preceded, or in the actual week, of sustaining a groin injury in elite under-age Australian footballers. Thus, it is possible that residual hip muscle fatigue due to limited recovery between successive matches could compromise the physical performance and lead to acutely reduced levels of hip adductor and abductor strength in field hockey players.

Therefore, the aim of this study was to examine the effects of two consecutive official field hockey matches, played on consecutive days, on selected risk factors for groin injury such as maximal hip adductor and abductor strength as well as self-perceived recovery status in female field hockey players. We hypothesized that there would be a progressive decline in the maximal hip adductor and abductor strength because the short time between successive matches would be insufficient to allow for complete neuromuscular recovery.

## Material and methods

### Participants

Fourteen healthy professional female field hockey players (age: 20 ± 5.4 years; body mass: 60.7 ± 7.2 kg; height: 167.0 ± 1.0 cm) took part in this prospective study. Participants were recruited from a Second Division field hockey team in Spain and they were training ~ 6.3 ± 0.8 h/week in the previous 3 months typically performing a single competitive match per week. Participants had an average of 11 ± 6 years of field hockey experience. Exclusion criteria were: (a) a history of adductor or abductor injury or related orthopaedic problems within the three previous months prior to testing; (b) impossibility to be tested due to other types of lower limb injury; (c) experiencing lower limb muscle soreness at time of baseline testing; (d) intake of medications or dietary supplements (e.g. caffeine) for the duration of the study. Goalkeepers were excluded due to the different nature of their activity during both training and match activities. Before taking part in the study, participants were fully informed about the experimental protocol and provided their written informed consent. This investigation was performed in accordance with the Declaration of Helsinki 2013 and was approved by the local Ethics Committee of the Universidad Francisco de Vitoria (45/2018).

### Data collection

**Fig. 1:**
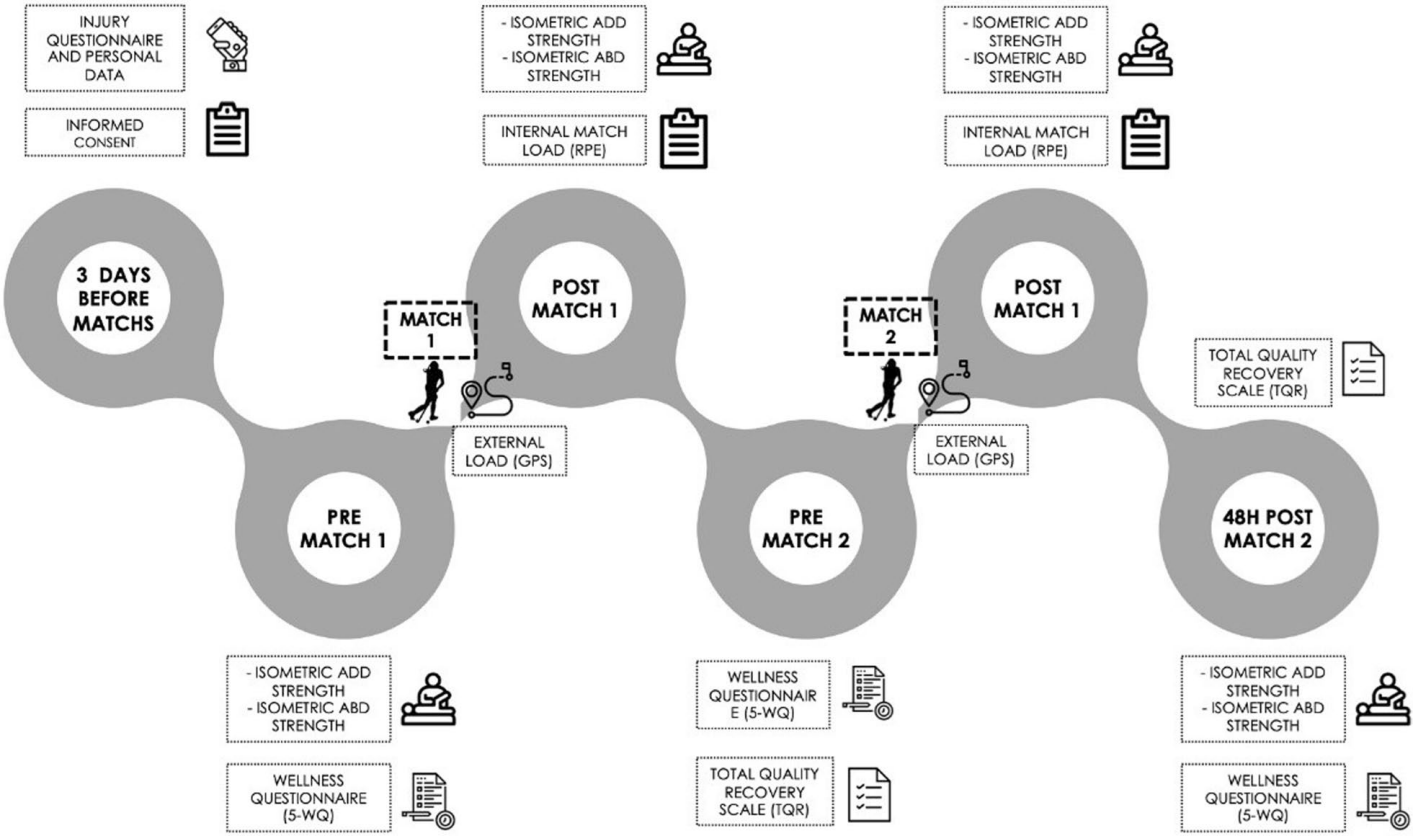
Study design of the measures obtained during the tournament

This study was a prospective and descriptive experiment that involved two official competitive field hockey matches played on consecutive days. Anthropometric data, age, medical history, training frequency, and years of experience were recorded for all participants before the start of the study. The matches were performed in November 2019 during a week that the field hockey team had two consecutive matches of the Spanish National League. Field hockey players were tested on four different occasions: before the first match (pre-match 1), immediately after the first match (post-match 1), after the second match (post-match 2), and again 48 h after the second match (48 h post-match 2; Fig. 1). Specifically, players were tested for maximal isometric hip adductor and abductor strength in both limbs 60 min pre-match 1 and immediately post-match 1, post-match 2, and 48 h post-match 2. According to previous procedures [18], the order of the tests and the selection of players were chosen at random. Limb dominance preference was defined as the leg used for kicking a ball determined by a questionnaire filled out by the participants [19]. Before pre-match testing, all players performed a five-minute warm-up including static stretches and joint mobility exercises [20]. All tests were conducted by three physiotherapists with several years of test experience. Moreover, the players completed the subjective Wellness Questionnaire (5-WQ) before pre-match 1, pre-match 2, and 48 h post-match 2 and Total Quality Recovery Scale (TQR) before pre-match 2 and 48 h post-match 2. Internal match load was reported using the session rate of perceived exertion (s-RPE) obtained from each player and it was measured 30 min after the end of the matches. Trunk-based accelerometer and Global Positioning System (GPS) units were used to record running and locomotion patterns during the matches. A month prior to baseline data collection, players performed a familiarization session to reduce the potential influence of learning effects on the outcomes of the investigation. The players were instructed to avoid alcohol and caffeine-containing foods/drinks for 24 h before and during the duration of the study.

### Measurements

#### Isometric hip adductor and abductor strength

Measurements of hip adductor and abductor isometric strength in both limbs were obtained with a hand-held dynamometer (Nicholas Manual Muscle Tester; Lafayette Indiana Instruments, Lafayette, IN, USA) according to the protocol by Thorborg et al. [20]. For this measurement, each participant lay supine on a stretcher, with their hips in a neutral position and stabilizing themselves by holding on to the sides of the stretcher. The examiner applied static resistance via a dynamometer placed 5 cm proximal to the proximal edge of the medial (for adductor) and lateral (for abductor) malleolus while the participants performed a 5-s maximum voluntary contraction against the dynamometer. Two repetitions were performed for both the dominant and non-dominant limbs, respectively, with a resting period of 30 s between successive. The best (highest peak force) of the two trials in the dominant and non-dominant limbs was selected to represent maximal hip adductor and abductor strength. Data were normalized by the body mass of the participant (N/kg).

#### Wellness questionnaire (5-WQ)

Subjective fatigue, sleep quality, general muscle soreness, stress levels, and mood were rated by each player using a validated five-point scale (5-WQ) by following the protocol proposed by McLean et al. [21]. Each question was scored by using a 1-to-5-point scale (0.5-point increase), with 1 and 5 representing poor and very good levels of well-being, respectively. The degree of “overall well-being” was determined by calculating the sum of the five scores.

#### Total Quality Recovery Scale (TQR)

To determine the self-perceived state of recovery, hockey players completed the TQR scale, as described by Kenttä et al. [22]. This scale ranges from 6 to 20 points, with 6 points being the minimum recovery value (i.e., representing zero or no recovery), and 20 points being the highest value of recovery (i.e., representing full, unconditional recovery).

#### External match load

The external match load was monitored using 10-Hz portable GPS and accelerometer units (Viper pod 2, STATSports, Belfast, Northern Ireland). The GPS units were placed in a neoprene harness on the players' backs between both scapulae. According to the manufacturer’s recommendations, all devices were activated 15-min before the initiation of data collection to allow the acquisition of satellite signals and synchronization of the GPS clock with the satellite’s atomic clock. The relative total distance (m/min) and the number of accelerations and decelerations above 3 m s^−2^ were recorded.

#### Internal match load

Internal match load was estimated using the session rating of perceived exertion (s-RPE [23]). The RPE value was determined using the Borg’s CR-10 scale, [ranging from “very, very easy” (1 point) to “maximal” (10-points)] recorded 30 min after the end of the match [23]. We obtained an s-RPE value for each match by multiplying the session intensity rating (RPE value) by the player’s involvement in the match (effective time of play, in minutes), to obtain an s-RPE value expressed in arbitrary units (A.U.) [24].

### Statistical analysis

Descriptive statistics (mean and standard deviation) were calculated for all variables obtained. Shapiro–Wilk test was used to assess the normal distribution of data. All variables presented a normal distribution in the test. Differences in s-RPE and running parameters, respectively, between the two matches, were evaluated using paired t-testing. A repeated-measures analysis of variance (ANOVA) was used to evaluate changes in isometric hip adduction and abduction strength across four different time points (pre-match 1, post-match 1, post-match 2, and 48 h after post-match 2). In case a significant time-effect was found, post-hoc testing was carried out subjected to Bonferroni correction. The effect sizes of the repeated measures ANOVA were measured using partial η^2^ values and the following thresholds were used: trivial (η^2^ ≤ 0.01); small (0.01 < η^2^ ≤ 0.06); medium (0.06 < η^2^ ≤ 0.14); and large (η^2^ > 0.14) [25]. A *post-hoc* power analysis was performed for the primary outcome variable considering large differences for effect size and the sample size, a power of 0.79 was obtained. The subjective variables obtained with the 5-WQ test were compared using non-parametric repeated measures Friedman’s analysis of variance (pre-match 1, pre-match 2, and 48 h after post-match 2). Post-hoc pairwise comparisons were done with the Durbin–Conover tests. The TQR was compared between pre-match 2 and 48 h after post-match 2 using paired t-testing. A confidence interval of 95% was determined for all analyses and statistical significance was set at *p* < 0.05. The statistical analysis was carried out using Jamovi (version 1.2.17; www.jamovi.org).

## Results

**Fig. 2:**
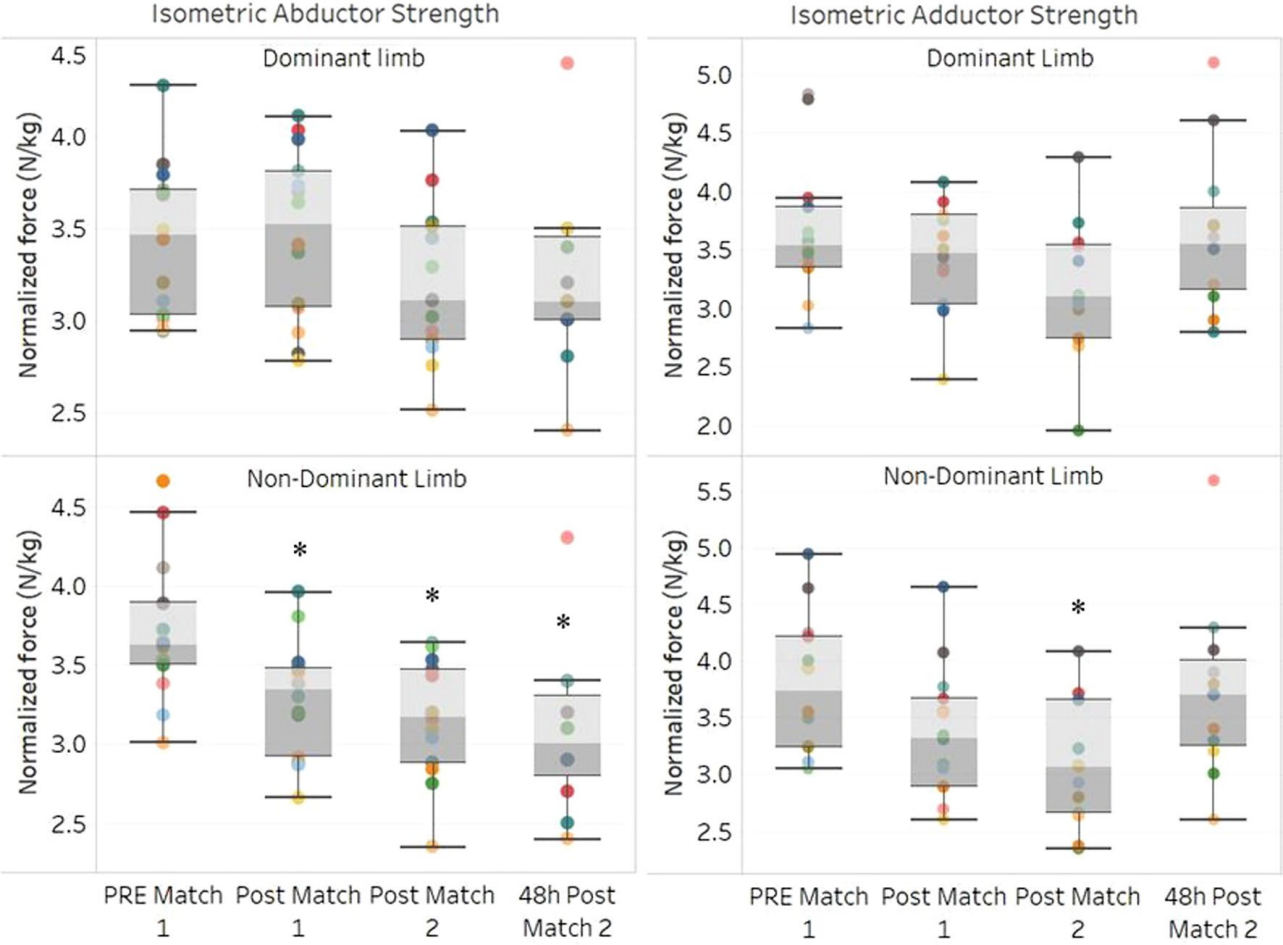
Isometric hip adductor and abductor strength in professional field hockey players performing two matches on two consecutive days. *Depicts a difference from pre-match 1, *p* < 0.05

Isometric hip muscle strength showed significant differences in the non-dominant limb for hip adduction (F (3,33) = 4.30, *p* = 0.011, partial η^2^ = 0.175, Fig. 2). The post-hoc tests showed a significant difference between pre-match 1 and post-match 2 values (p_bonferroni_ = 0.014), while no other significant differences were found between any time points. For the dominant limb, no difference in time effect was observed (F (3,33) = 2.79, *p* = 0.056, partial η^2^ = 0.119), but no significant difference was found between successive time points of measurement.

In the case of isometric hip abductor strength, a time-effect was observed for the non-dominant limb (F (3,33) = 4.30, *p* < 0.001, partial η^2^ = 0.312), with post-hoc tests revealing a decrease at post-match 1 (p_bonferroni_ = 0.034), post-match 2 (p_bonferroni_ = 0.002) and 48 h after post-match 2 (p_bonferroni_ < 0.001) compared to pre-match 1 values. In contrast, no time-effect was observed for the dominant limb time-effect (F (3.33) = 1.52, *p* = 0.227, η^2^ = 0.081).

No significant changes were observed between the two matches for any of the locomotion variables investigated. The physical performance variables showed no significant differences between the matches (Table 1).

**Table 1:** Physical performance variables and s-RPE values reported for the two matches

	Match 1	Match 2	*p *value
Relative total distance covered (m min^−1^)	149.40 ± 80.10	153.47 ± 44.57	0.917
Number of accelerations above 3 m s^−2^	10.23 ± 8.22	11.00 ± 6.47	0.785
Number of decelerations exceeding 3 m s^−2^	4.31 ± 3.77	5.62 ± 4.56	0.083
s-RPE	6.73 ± 1.90	7.21 ± 2.12	0.121

*AU* arbitrary units

Self-perceived wellness measured by 5-WQ (Table 2) remained unchanged between the three-time points of measurement (χ^2^ = 2.00, df = 2, *p* = 0.368). However, a significant time effect was observed for fatigue rating (χ^2^ = 7.15, df = 2, *p* = 0.028), with players experiencing more fatigue on pre-match 2 compared to pre-match 1 (*p* = 0.009), while players recovered back to pre-competition levels at 48 h post-match 2 (*p* = 0.027). No difference in time-effect was observed for the muscle soreness parameter (χ^2^ = 5.82, df = 2, *p* = 0.055), with significant differences emerging between pre-match 1 and pre-match 2 (*p* = 0.015). The remaining parameters showed no differences between the three-time points of data collection. Similarly, no differences were found in TQR between pre-match 2 and 48 h after post-match 2 (t = 1.57, *p* = 0.142).

**Table 2:** Items obtained in the subjective wellness questionnaire (5-WQ) in professional field hockey players competing in two matches on consecutive days

(5-WQ)	Pre-match 1	Pre-match 2	48 h post-match 2	Pre-match 1 versus pre-match 2	Pre-match 1 versus 48 h post-match 2	Pre-match 2 versus 48 h post-match 2
				*p*	*p*	*p*
Fatigue	3.79 ± 0.89	3.21 ± 1.05	3.75 ± 0.75	0.009	0.639	0.027
Sleep	3.79 ± 0.70	3.79 ± 0.80	3.58 ± 0.90	1.000	0.374	0.374
Soreness	3.64 ± 0.63	3.14 ± 0.86	3.42 ± 0.51	0.015	0.198	0.198
Stress	3.79 ± 0.80	3.79 ± 0.97	3.50 ± 1.17	0.810	0.904	0.904
Mood	4.00 ± 0.88	4.36 ± 0.63	4.00 ± 0.74	0.243	0.882	0.306
Total wellness	19.00 ± 2.66	18.29 ± 3.00	18.25 ± 2.93	0.232	0.232	1.000

## Discussion

The primary aim of this study was to examine the accumulated effect of successive official field hockey matches, played on consecutive days on selected risk factors for groin injury (i.e., maximal hip adductor and abductor strength). The secondary aim was to analyze the locomotive demands (i.e., GPS) and self-perceived exertion and recovery parameters in female competitive field hockey players. The main findings of this investigation indicate that abductor and adductor muscle strength in the non-dominant limb was reduced in comparison to pre-competition values, suggesting that muscle weakness accumulated during the two consecutive matches. Notably, match performance (locomotion profile) in the second match was similar to the first match. This may indicate that professional field hockey players are at a higher risk of groin injury when exposed to successive matches on consecutive days, although match performance may not be substantially affected by the accumulation of fatigue. From a practical application view, physiotherapist/strength and conditioning coaches could monitor maximal isometric hip strength during congested calendars to assess the likelihood of sustaining a groin injury [6].

To our best knowledge, no previous investigation has examined the effects of consecutive match play on maximal hip adductor muscle strength in field hockey players. However, previous research has demonstrated that hip adductor muscle strength is significantly reduced after football matches played consecutively with insufficient recovery (≤ 96 h of rest between matches) compared to baseline levels [17, 26]. Thus, in line with previous reports, the present data revealed that isometric adductor strength decreased (− 10.6%) acutely after the first match (post-match 1) in the non-dominant limb and after the second match (post-match 2) further decreased both in the non-dominant (− 17.5%) and dominant limbs (− 13.4%) when compared to pre-match 1 levels.

The acute decrements in maximal hip strength observed following match-play may potentially be explained by onset of neuromuscular fatigue [27] imposed by the specific physical demands of field hockey play. In support of this notion, field hockey players perform a high number of short high-intensity accelerations, decelerations, and rapid changes in direction including side-cutting during matchplay [13], which are likely to require very high concentric and eccentric contraction forces by the hip adductor muscles. In addition, it is well known that short congestion periods of high loads in field hockey may increase muscle damage markers (i.e., creatine kinase) and residual fatigue that could require a rest period to recovery previous fitness levels [28]. Assuming a similar match load for competitive field hockey players, this may explain the augmented decrease in hip adductor strength observed at post-match 2 in the present study. Consequently, the acute and sustained reductions in maximal hip adductor muscle strength presently observed for the non-dominant side (10.6–17.5%) following consecutive days of match play might represent a significant risk factor for the development of groin injury, especially in players most prone to injury (indicated by low strength levels of the hip adductor and restricted hip ROM [16].

According to previous reports, reduced strength of hip abductor muscles may affect the normal kinematics of the hip joint. Specifically, hip abductor weakness may increase the range of internal hip rotation and knee abduction, respectively, subsequently increasing the risk of injury including non-contact ACL rupture during sport [29, 30]. Therefore, monitoring hip abduction muscle strength could be useful to control the injury risk associated with acute or long-term reductions in this variable. In the present study, maximal hip abductor strength was found to decrease in the non-dominant limb when comparing post-match 1 (− 11.1%), post-match 2 (− 14.9%) and 48 h post-match 2 (− 17.0%) levels to baseline (pre-match 1). Since no previous study has analyzed hip isometric abductor muscle strength in field hockey players, comparisons to the literature are not possible. A possible reason for the decrease in hip abduction strength observed at post-match 2 on the non-dominant side could be related to sarcomere disruptions within the muscular fibres due to the repeated overextension actions that occur during the different field hockey movements (e.g. accelerations, deceleration, change of directions) [31] and/or accumulating fatigue. It is well known that the diverse movement patterns and joint activities involved with field hockey require vigorous involvement of both the dominant and non-dominant lower limbs, for example during the repetitive unidirectional pivoting movements performed during forehand strokes and drag-flicks (i.e., preferred method of scoring during penalty corners in field hockey, mentioning that small number of players are specialized players in drag flicking actions) [32]. These strokes and player actions involve a combination of powerful trunk rotations, high muscle force production, and synchronization of lower limb movements [33] that altogether may predispose for overuse injuries (e.g. hip or lower back) not least due to the high volume of skill-specific training required to develop and maintain expertise in drag flicking [34]. In addition, a recent biomechanical study has shown that field hockey actions (such as above) characterized by hip flexion performed near the end range of motion (i.e. highly flexed) combined with hip joint rotation and abduction may contribute to an increased prevalence of hip injuries in field hockey players [35].

Very few studies have examined the relationship between congested match demands and mechanical muscle function in field hockey players. McGuinness and coworkers (2018) examined the physical state and physiological capacity of elite field hockey players during a condensed tournament program where seven matches were played in less than 2 weeks [36]. Notably, high intensity running during the matches progressively decreased throughout the tournament, with lower distances covered in games five, six, and seven when compared to game one [36]. The present results showed that running activity (i.e., distance covered and speed of movement, in many team sports) could be sustained in the second game, in spite of the fact that there was less than one day of recovery. Consequently, despite physiological impairments in hip muscle strength caused by two field hockey matches played on consecutive days, this did not lead to measurable decrements in playing performance (distance covered, acceleration-deceleration profiles). It is possible, however, that performance impairments would occur in the case of a longer tournament with a higher number of consecutive matches.

Measures of subjective wellness have been considered a useful tool to quantify and report the internal response to given activities of physical loading [37]. In the current study, female hockey players showed elevated indices of fatigue and muscle soreness 48 h after pre-match 2 in comparison with baseline levels (pre-match 1). No temporal changes were observed for any other variables included in the 5-WQ analysis. Accumulating fatigue and muscle soreness as well as signs of impaired subjective wellness may generally be explained by the engagement in repeated match demands [26]. The present results support the use of subjective wellness monitors such as the 5-WQ tool to evaluate mental recovery during periods of condensed and congested match play, as it could provide insightful information to coaches to individualize recovery strategies and to manage training load exposure in the preparation for subsequent matches.

In terms of perceived exertion of the players, the present study reported similar s-RPE (346.1 ± 231.7 AU in match 1 and 392.31 ± 203.82 AU in match 2) when compared to previous reports [34] (348 ± 61 AU in match 1 and 436 ± 85 AU in the match 2). In addition, McGuinness et al. [38] reported significant increases in s-RPE between match 1 and match 2, which was supported by a strong tendency (*p* = 0.056) for an increase in-s-RPE following match 2 in comparison with pre–post-match 1. Considering that it is common for professional hockey players to participate in consecutive daily matches during packed tournaments as part of their competition schedule, further research could prove useful to identify the relationship between physical demands imposed during consecutive matches in field hockey players and the resulting changes in s-RPE [39].

As for self-perceived recovery (TQR), no significant changes were found in the present study. Since there are no previous studies in hockey analyzing the perceived recovery via TQR in field hockey players, comparisons with the literature were not possible. The present lack of changes in TQR is partially supported by previous observations in other intermittent-type team sports such as football Gjaka et al. [39], with players engaging in either one or two matches per week over a 4-week period. Collectively, the available data thus suggest TQR to be less sensitive than wellness measures in the monitoring of field hockey players during the time course of condensed match programs.

## Limitations

Some potential limitations may be recognized for the experimental design used in this investigation. Firstly, the present study participants represented a specific sample of female field hockey players, and the present findings may therefore not extend to goalkeepers, younger players, male field hockey players nor to athletes from other team sports. In addition, the results of this investigation are specific to the field hockey competition entailing two consecutive matches while other competitive scenarios with a higher number of matches or higher recovery periods may present different results. Further, the match-induced changes in hip muscle strength found in the present investigation were not directly compared against the actual incidence of groin injuries because the duration of the investigation was insufficient to obtain a representative number of groin injuries/complaints. Hence, the outcomes of this investigation should be confirmed with more prolonged experiments, where the development of groin injuries can be compared to fluctuations in maximal hip abductor and adductor muscle strength. Last, although hip muscle weakness, especially for the hip adductors, appears to represent a recurrent finding in previous investigations of risk factors for groin injury in team sports [7–9] the present observations of acute and more prolonged (+ 48 h) hip muscle weakness do not per se entail the development of groin injury.

## Conclusions

In summary, isometric hip adductor and abductor strength in the non-dominant limb were reduced at post-match 1 and post-match 2 compared to baseline (pre-match 1) levels. These observations suggest that two consecutive matches of field hockey, played on consecutive days, may increase the risk of groin injury in professional female players due to transient (+ 48 h) hip abductor weakness. In addition, the current study showed signs of enhanced fatigue and muscle soreness at pre-match 2 compared to pre-match 1, while fatigue was recovered 48 h after the second match (5-WQ). These outcomes point toward a progressive accumulation in fatigue during densely packed tournaments, although not affecting running performance when assessed in match 2. Collectively, the current data underline the necessity of monitoring the hip adductor and abductor strength and the fluctuations in players’ wellness in hockey competing in a congested match program. These routines may help identify field hockey players with excessive fatigue, who may be prone to groin injury due to acute and persistent (+ 48 h) hip muscle weakness.

## Data Availability

Full data for this research is available through the corresponding author upon request.

## References

[1] Barboza SD, Joseph C, Nauta J, van Mechelen W, Verhagen E (2018). Injuries in field hockey players: a systematic review. Sports Med.

[2] Whittaker JL, Small C, Maffey L, Emery CA (2015). Risk factors for groin injury in sport: an updated systematic review. Br J Sports Med.

[3] Bisciotti GN, Auci A, Di Marzo F, Galli R, Pulici L, Carimati G, Quaglia A, Volpi P (2015). Groin pain syndrome: an association of different pathologies and a case presentation. Muscles Ligaments Tendons J.

[4] Ibrahim A, Murrell GAC, Knapman P (2007). Adductor strain and hip range of movement in male professional soccer players. J Orthop Surg.

[5] Tak I, Glasgow P, Langhout R, Weir A, Kerkhoffs G, Agricola R (2016). Hip range of motion is lower in professional soccer players with hip and groin symptoms or previous injuries, independent of cam deformities. Am J Sports Med.

[6] Crow JF, Pearce AJ, Veale JP, VanderWesthuizen D, Coburn PT, Pizzari T (2010). Hip adductor muscle strength is reduced preceding and during the onset of groin pain in elite junior Australian football players. J Sci Med Sport.

[7] Barboza SD, Nauta J, van der Pols M, van Mechelen W, Verhagen E (2018). Injuries in Dutch elite field hockey players: a prospective study. Scand J Med Sci Sports.

[8] Moreno-Pérez V, Lopez-Valenciano A, Barbado D, Moreside J, Elvira JLL, Vera-Garcia FJ (2017). Comparisons of hip strength and countermovement jump height in elite tennis players with and without acute history of groin injuries. Musculoskelet Sci Pract.

[9] Moreno-Pérez V, Travassos B, Calado A, Gonzalo-Skok O, Del Coso J, Mendez-Villanueva A (2019). Adductor squeeze test and groin injuries in elite football players: a prospective study. Phys Ther Sport.

[10] Ryan J, DeBurca N, Mc Creesh K (2014). Risk factors for groin/hip injuries in field-based sports: a systematic review. Br J Sports Med.

[11] Tyler TF, Nicholas SJ, Campbell RJ, McHugh MP (2001). The association of hip strength and flexibility with the incidence of adductor muscle strains in professional ice hockey players. Am J Sports Med.

[12] Dick R, Hootman JM, Agel J, Vela L, Marshall SW, Messina R (2007). Descriptive epidemiology of collegiate women's field hockey injuries: National collegiate athletic association injury surveillance system, 1988–1989 through 2002–2003. J Athl Train.

[13] Singh J, Appleby BB, Lavender AP (2018). Effect of plyometric training on speed and change of direction ability in elite field hockey players. Sports.

[14] Nedelec M, McCall A, Carling C, Legall F, Berthoin S, Dupont G (2012). Recovery in soccer: part I—post-match fatigue and time course of recovery. Sports Med.

[15] Romero-Moraleda B, Morencos-Martínez E, Torres-Londa L, Casamichana D (2020). Analysis of congested schedule on competition external load in field hockey. RICYDE Rev Int Cienc Deporte.

[16] Wollin M, Pizzari T, Spagnolo K, Welvaert M, Thorborg K (2018). The effects of football match congestion in an international tournament on hip adductor squeeze strength and pain in elite youth players. J Sports Sci.

[17] Wollin M, Thorborg K, Pizzari T (2018). Monitoring the effect of football match congestion on hamstring strength and lower limb flexibility: potential for secondary injury prevention?. Phys Ther Sport.

[18] Sánchez-Migallón V, López-Samanes Á, Terrón-Manrique P, Morencos E, Fernández-Ruiz V, Navandar A, Moreno-Pérez V (2020). The acute effect of match-play on hip isometric strength and flexibility in female field hockey players. Appl Sci.

[19] Thorborg K, Bandholm T, Petersen J, Weeke KMØ, Weinold C, Andersen B, Serner A, Magnusson SP, Hölmich P (2010). Hip abduction strength training in the clinical setting: with or without external loading?. Scand J Med Sci Sports.

[20] Thorborg K, Petersen J, Magnusson SP, Hölmich P (2010). Clinical assessment of hip strength using a hand-held dynamometer is reliable. Scand J Med Sci Sports.

[21] McLean B, Coutts A, Kelly V, McGuigan M, Cormack S (2010). Neuromuscular, endocrine, and perceptual fatigue responses during different length between-match microcycles in professional rugby league players. Int J Sports Physiol Perform.

[22] Kenttä G, Hassmén P (1998). Overtraining and recovery. A conceptual model. Sports Med.

[23] Foster C, Florhaug JA, Franklin J, Gottschall L, Hrov LA, Suzanne P, Doleshal A, Dodge C (2001). A new approach to monitoring exercise training. J Strength Cond Res.

[24] Impellizzeri FM, Rampinini E, Coutts AJ, Sassi A, Marcora SM (2004). Use of RPE-based training load in soccer. Med Sci Sports Exerc.

[25] Cohen J (1992). Quantitative methods in psychology. Psychol Bull.

[26] Silva JR, Rumpf MC, Hertzog M, Castagna C, Farooq A, Girard O, Hader K (2018). Acute and residual soccer match-related fatigue: a systematic review and meta-analysis. Sports Med.

[27] Thorlund JB, Aagaard P, Madsen K (2009). Rapid muscle force capacity changes after soccer match play. Int J Sports Med.

[28] Walker AJ, McFadden BA, Sanders DJ, Bozzini BN, Conway SP, Arent SM (2020). Early season hormonal and biochemical changes in division I field hockey players: is fitness protective?. J Strength Cond Res.

[29] Bencke J, Aagaard P, Zebis MK (2018). Muscle activation during ACL injury risk movements in young female athletes: a narrative review. Front Physiol.

[30] Nakagawa TH, Moriya ETU, MacIel CD, Serrão FV (2012). Trunk, pelvis, hip, and knee kinematics, hip strength, and gluteal muscle activation during a single-leg squat in males and females with and without patellofemoral pain syndrome. J Orthop Sports Phys Ther.

[31] Proske U, Morgan DL (2001). Muscle damage from eccentric exercise: mechanism, mechanical signs, adaptation and clinical applications. J Physiol.

[32] López De Subijana C, Gómez M, Martín-Casado L, Navarro E (2012). Training-induced changes in drag-flick technique in female field hockey players. Biol Sport.

[33] Ng L, Sherry D, Loh WB, Sjurseth AM, Iyengar S, Wild C, Rosalie S (2016). The prevalence and severity of injuries in field hockey drag flickers: a retrospective cross-sectional study. J Sports Sci.

[34] Ng L, Rosalie SM, Sherry D, Loh WB, Sjurseth AM, Iyengar S, Wild CY (2018). A biomechanical comparison in the lower limb and lumbar spine between a hit and drag flick in field hockey. J Sports Sci.

[35] Beddows TPA, van Klij P, Agricola R, Tak IJR, Piscaer T, Verhaar JAN, Weir A (2020). Normal values for hip muscle strength and range of motion in elite, sub-elite and amateur male field hockey players. Phys Ther Sport.

[36] McGuinness A, Malone S, Hughes B, Collins K (2018). Physical activity and physiological profiles of elite international female field hockey players across the quarters of competitive match-play. J Strength Cond Res.

[37] Gastin PB, Meyer D, Robinson D (2013). Perception of wellness to monitor adaptive response to training and competition in elite Australian football. J Strength Cond Res.

[38] McGuinness A, McMahon G, Malone S, Kenna D, Passmore D, Collins K (2020). Monitoring wellness, training load, and running performance during a major international female field hockey tournament. J Strength Cond Res.

[39] Gjaka M, Tschan H, Francioni FM, Tishkuaj F, Tessitore A (2016). Monitoring of loads and recovery perceived during weeks with different schedule in young soccer players. Kinesiol Slov.

